# The Role of NPY in the Regulation of Bone Metabolism

**DOI:** 10.3389/fendo.2022.833485

**Published:** 2022-02-22

**Authors:** Qing-Chang Chen, Yan Zhang

**Affiliations:** ^1^ Department of Ultrasound Medicine, Union Hospital, Tongji Medical College, Huazhong University of Science and Technology, Wuhan, China; ^2^ Hubei Province Key Laboratory of Molecular Imaging, Wuhan, China; ^3^ Department of Pediatrics, Union Hospital, Tongji Medical College, Huazhong University of Science and Technology, Wuhan, China

**Keywords:** bone disease, NPY, bone formation, bone resorption, osteoporosis

## Abstract

Bone diseases are the leading causes of disability and severely compromised quality of life. Neuropeptide Y (NPY) is a multifunctional neuropeptide that participates in various physiological and pathological processes and exists in both the nerve system and bone tissue. In bone tissue, it actively participates in bone metabolism and disease progression through its receptors. Previous studies have focused on the opposite effects of NPY on bone formation and resorption through paracrine modes. In this review, we present a brief overview of the progress made in this research field in recent times in order to provide reference for further understanding the regulatory mechanism of bone physiology and pathological metabolism.

## Introduction

The mammalian skeleton is a vital organ formed by several bone types, and it is also the place for hematopoiesis and mineral storage, with powerful self-repair ability and mineralized extracellular matrix. The traditional view of factors affecting bone metabolism such as endocrine, paracrine, and mechanical stimulation has long been discussed. Recent findings reported that bone tissue (including the periosteum, cortical and trabecular bone, bone marrow) was abundantly innervated by autonomic nerve terminals, which is one of the key factors regulating bone metabolism and remodeling through direct or indirect manner (1, 2), making the autonomic nerve system and bone metabolism closely linked.

When neuropeptide Y (NPY) was first discovered in 1983, the awareness of its function in energy balance, obesity, and bone metabolism has gradually increased (3, 4). As a 36-amino acid peptide belonging to the pancreatic polypeptide family, NPY is most abundantly produced and expressed in the nervous system (5). In the central nervous system, NPY is distributed in the amygdala, locus coeruleus, and cerebral cortex, with the highest expression level in the hypothalamus. It acts to coordinate signals from a wide variety of sources to participate in appetite, circadian rhythm, and energy utilization regulation (6, 7). In the periphery, NPY was found to be co-stored and co-released with neurotransmitter noradrenaline (NA) in postganglionic sympathetic nerves (8). Recent studies have reported that NPY and its receptors have also been identified in bone tissue, such as in osteoblasts, osteocytes, and adipocytes (2, 9, 10), indicating the potential role of NPY on bone remodeling in local sites. Moreover, it can also act as a mediator of the autonomic nervous system to mediate bone marrow mesenchymal cell (BMSC) differentiation fate by constructing a mouse model that lacks osteocyte-specific NPY (2). Even though various physiological conditions and pathophysiological processes such as obesity (11), anxiety (12), food intake (13), chronic pain (14), neurodegenerative disorders (15), and bone disease (2) have been proven to require NPY to participate, its effect on bone metabolism is still poorly understood.

In this review, we focus on the effects of NPY on bone metabolism in some physiological and pathological states. The aims of this article are to review the regulatory effects and to achieve a comprehensive understanding of NPY on bone metabolism.

## Neuropeptide Y and Its Receptors

Bone remodeling involves mineralized bone removal by osteoclasts followed by bone matrix formation through osteoblasts that subsequently become mineralized (16). It is a key process for maintaining bone mass in a dynamic balance and continues throughout life. Previous studies have proven the vital role of NPY in the regulation of food intake and energy homeostasis, and its role in bone metabolism has gradually become a hot topic in recent years.

NPY is a highly conserved endogenous peptide and multifunctional neurotransmitter acting *via* five G-protein-coupled receptor subtypes named Y1R, Y2R, Y4R, Y5R, and Y6R, of which Y1R and Y2R modulate bone mass at differing sites and through different ways (2, 14, 17). The arcuate nucleus of the hypothalamus exhibited the greatest expression level of NPY, and Y2R is the most abundant subtype in the central nervous system (18), which is also peripherally found in the liver, intestine, spleen, muscle, and adipose tissue, suggesting Y2R may have local effects in these tissues (19). Y2 antagonist treatment resulted in reduced bone resorption level and greater bone mineral density in ovariectomized (OVX) mice (20). Hypothalamic Y2R knockout mice exhibited increased osteoblast activity, mineralization rate, and bone mass, indicating a catabolic role of Y2R in stimulating cortical and cancellous bone formation (**Table 1**) (28, 29).

**Table 1:** Characterization, distribution, and functions of NPY receptors.

Receptor	Tissue distribution	Physiological functions on bone	Other functions	Ref.
Y1R	Hypothalamus, hippocampus, neocortex, thalamus, bone cells, pancreas, intestine	BMSC proliferation, osteogenic and adipogenic differentiation, macrophage migration, regulated gut microbiota, pulpal development	Vasoconstriction, anxiolysis, food intake, heart rate, anxiety	(17, 21–27)
Y2R	Hippocampus, hypothalamus, brain stem, articular cartilage, liver, intestine, spleen, muscle, and adipose tissue	Osteoblast activity and mineralization rate, cartilage homeostasis	Memory, circadian rhythm, angiogenesis, epilepsy	(10, 19, 20, 28–30)
Y4R	Total brain, heart, thoracic aorta, coronary artery, nasal mucosa, skeletal muscle, mesentery vasculature, stomach, ileum, and endometrium	Synergize with Y2R	Energy expenditure, anxiety-like and depression-related behavior, ion transportation, arterial pressure	(31–33)
Y5R	Hypothalamus, hippocampus	BMSC proliferation	Food intake, epilepsy, circadian rhythm	(34–36)
Y6R	Hypothalamus	Osteoblast precursor survival and Osteoclast activity	food intake	(37, 38)

Y1R has also been reported to be involved in many physiological activities, such as mitogenic activity, macrophage migration, and pulpal development (17, 21, 22). In bone tissue, Y1R is highly expressed in BMSCs, osteoblast, osteocyte, monocyte/macrophage, and osteoclast (2), prompting it to play a regulatory role in the local area. Y1R germline deletion resulted in elevated osteoblast activity and mineral apposition rate, together with increased formation of highly multinucleated osteoclasts and enhanced surface area, demonstrating a negative role of Y1R on bone mass maintenance (23, 24). Furthermore, the Y1R antagonist regulated gut microbiota and exhibited an anti-osteoporotic effect in OVX rats (25), revealing that Y1R may affect bone mass through multiple ways.

To date, little is known about the role of Y4R, Y5R, and Y6R in bone mass maintenance. Y4R was reported to mainly affect body weight, fat mass, energy expenditure, and anxiety-like and depression-related behavior (31, 32). Interestingly, male mice lacking both Y2R and Y4R displayed a synergistic effect in trabecular bone volume upregulation compared with Y2R knockout mice, but female double knockout mice did not show this bone phenotype, suggesting a synergy between Y2 and Y4 receptor pathways (33). Igura et al. reported that Y5R expression level in bone marrow cells declined with age and Y5R overexpression strengthened the proliferation effect induced by NPY, indicating that Y5R may take part in bone metabolism by affecting the self-renewal ability of bone marrow cells (34). Y6R, which is restricted to the suprachiasmatic nucleus (SCN) of the hypothalamus, is required for the maintenance of bone mass in mice. Mice lacking Y6R displayed reduced numbers of osteoblast precursors and increased osteoclast activity (37).

## NPY and Bone Formation

As seed cells in bone marrow, BMSCs are able to commit to osteogenic lineage and differentiate into mature osteoblasts. Intensive studies in recent years have demonstrated that a number of transcription factors are involved in this process. Among them, runt-related transcription factor 2 (runx2) and osterix are considered as master transcription factors in osteogenic differentiation and they control bone formation (39). Zhang et al. found that runx2 level and mineralized nodules were decreased after NPY treatment in osteogenic differentiation of BMSCs, confirming that NPY inhibits osteogenesis by inhibiting runx2, and this effect may be achieved through Y1R (2). Germline deletion of Y1R and knockout of NPY produce anabolic responses in bone, with upregulated runx2 and osterix level, resulting in a generalized increase in bone mass owing to stimulated osteoblast activity and an increased bone formation rate (40, 41). Besides, dorsomedial nucleus NPY knockdown mice showed increased basal and obesity-induced decrease in bone mineral density (BMD) together with reduced activating transcription factor 4 (ATF4) expression level (42). Activator protein 1 (AP1) antagonists targeted to NPY neurons resulted in increased trabecular bone formation and mass (43). In glucocorticoid-induced osteoporotic skeleton, NPY expression and marrow adipogenesis were upregulated, together with increased post-translational modification of peroxisome proliferator-activated receptor gamma (PPARγ) (44).

Paradoxically, several studies have reported that NPY acts as a promoting factor in the process of bone formation and fracture repair. Liu et al. found that low doses of NPY stimulate BMSC osteogenic differentiation and mineralization while a high NPY concentration had the opposite effect (45). In patients with combined injuries, NPY levels were increased than in those with simple fractures, and further experiment demonstrated that NPY directly promotes BMSC osteogenic differentiation (46). Y1R antagonist-treated mice or Y1R-deficient mice exhibited a delay in fracture repair and cartilage removal, as evidenced by reduced calcified nodule area and decreased bone callus volume and strength (47, 48). Researchers recently used overexpression plasmids and small interfering RNA (siRNA) targeting NPY transfected into the MC3T3−E1 osteoblastic cell line and found that NPY overexpression markedly enhanced the osteogenic ability by an autocrine mechanism, together with the upregulation of osterix and runx2 level (49). Knockdown of the Y1R induced alkaline phosphatase (ALP) activity and mineralization together with upregulated mRNA expression of specific genes that characterize osteoblastic differentiation in MC3T3−E1 cells (50).

As an anxiolytic factor, NPY was reported to protect against chronic stress‐induced bone loss specifically through Y2R, evidenced by increased bone mass and bone formation rate (51). Also, NPY can regulate bone formation through an indirect manner. Ma et al. found that NPY stimulated human osteoblast osteogenic activity by enhancing gap junction intercellular communication (52). The Y1R antagonist upregulated serum Ca^2+^ concentration, changed the gut microflora community composition, and improved bone mass in OVX rats (25). Although the studies mentioned above seem inconsistent, it is certain that bone formation is strongly influenced by NPY.

## NPY and Bone Resorption

Bone resorption was mediated by mature osteoclast, which is a tissue-specific multinuclear giant cell derived from hematopoietic stem cells through the myelomonocytic precursor cells/macrophage lineage. In brief, hematopoietic stem cells are committed to macrophage colony-forming units (CFU-M) in the presence of macrophage colony-stimulating factor (M-CSF). When the receptor activator of nuclear factor-kappa B ligand (RANKL) binds RANK on the surface of osteoclast precursors, osteoclastogenesis is immediately triggered. CFU-M is further differentiated into mononucleated osteoclasts and subsequently fused to multinucleated osteoclasts, then fully matured upon a cognate interaction with osteoblasts (53). Wu et al. reported that NPY greatly increased the amount of RAW264.7 cell (mouse leukemic monocyte macrophage cell line) migration at different concentrations, and this effect can be diminished by the Y1R antagonist and ERK1/2 inhibitor, which suggest that NPY promotes osteoclast migration through Y1R and ERK1/2 activation (22). NPY has also been shown to exhibit an inhibitory effect on isoprenaline-induced osteoclastogenesis by suppressing RANKL expression in mouse bone marrow cells (54). In addition, an *in-vitro* experiment confirmed that the regulator of osteoclastogenesis RANKL/OPG ratio was higher in NPY-treated BMSCs, and this effect can be reversed with Y1R antagonist treatment, making evidence that NPY may facilitate bone resorption through Y1R (55).

On the contrary, Park et al. found that NPY can mobilize hematopoietic stem/progenitor cells (HSPCs) from the bone marrow to the peripheral blood and ameliorated low bone density in an ovariectomy-induced osteoporosis mouse model by reducing osteoclast number (56). Seldeen et al. used an osteoporotic mouse model injected once daily with JNJ-31020028, a brain-penetrant Y2R small molecule antagonist. Then, primary bone cell cultures were isolated from the tibiae, and it was found that bone marrow cultures obtained from the Y2R antagonist-treated mice exhibited significantly more osteoclasts and greater areal coverage with *in-vitro* osteoclast differentiation induction, which means that central NPY inhibited osteoclastogenesis through Y2R (20).

In our study, osteoclast number and activity seem not be significantly influenced by bone-specific deficiency of NPY in young and aged mice (2). Matic et al. generated a mouse model where NPY was overexpressed specifically in mature osteoblasts and osteocytes and characterized the bone phenotype of 3-month-old mice. It was found that bone volume was reduced; however, bone formation rate and osteoclast activity were not significantly changed (57). The direct and indirect effects of NPY on bone resorption need further exploration.

## Others

**Figure 1:**
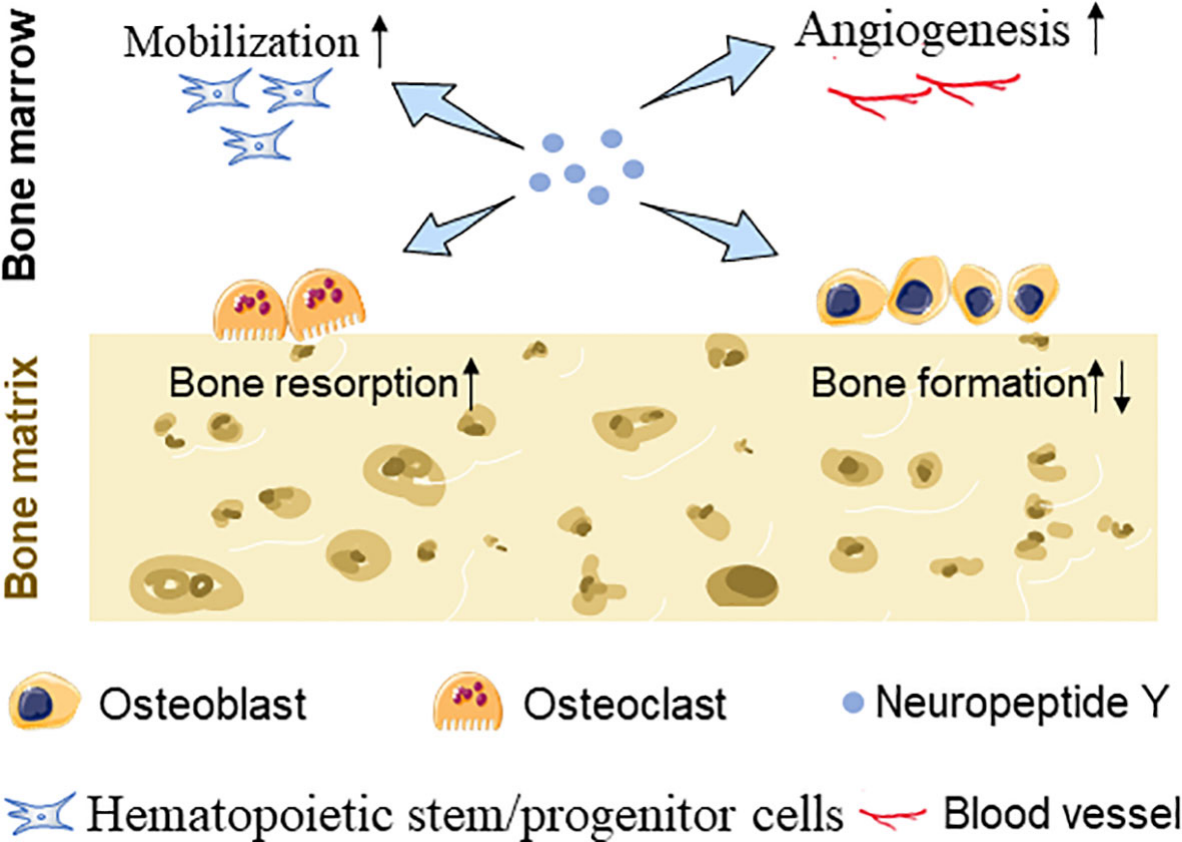
Schematic diagram showing NPY-mediated BMSC mobilization, EC angiogenesis, and bone turnover changes.

In addition to participating in bone metabolism through affecting bone turnover, NPY may also affect bone mass through other ways. Blood vessels play an irreplaceable important role in the metabolic balance of bones. Several studies have confirmed that NPY-immunoreactive fibers were predominantly localized alongside with blood vessel walls in bone; moreover, Y1R, Y2R, and Y5R were confirmed to be expressed on endothelial cells (ECs), providing a material basis for the vasoregulatory role of NPY in addition to directly regulating bone tissue cells (58). It has been observed that BMSC migration and VEGF expression were upregulated after NPY treatment (45) and increased levels of VEGF stimulate angiogenesis and osteoblastic differentiation of BMSCs (59). Besides, Y1R signaling disruption is responsible for enhancing the deposition and maturity of collagen and mineral hydroxyapatite layers in the skeletal muscle, and bone mechanical property was furthered improved (60) (**Figure 1**).

## Relationship of NPY and Common Bone Disease

### Osteoporosis

Osteoporosis (OP) is a common skeletal disorder characterized by compromised bone mass and degraded bone microarchitecture, often resulting in fragility fractures and severely compromised quality of life in elderly people. Increasing age and postmenopausal state are proven to be associated with this condition. Zhang et al. reported that ovariectomy induced NPY upregulation in bone tissue after constructing a model of OP in adult female mouse. γ‐Oryzanol (ORZ), a functional substance extracted from rice bran, alleviated the severity of postmenopausal and senile OP through the autonomic nervous system by inhibiting osteocytic-NPY secretion (2). In glucocorticoid-mediated bone loss, NPY mRNA expression and protein concentration were elevated, while BMD and bone microstructure were significantly reduced (44). Xie et al. reported that the OP group exhibited deteriorated bone microstructure and more microdamage than the osteoarthritis (OA) group, and they also measured NPY and Y1R expression levels in patients after constructing a postmenopausal osteoporotic rat model and found these to be both upregulated in OP groups. Y1R antagonist treatment *in vivo* for OVX rats could improve bone microstructure and decrease bone microdamage, and this may be achieved *via* the cAMP/PKA/CREB signaling pathway (10). Also, NPY is increased in the rat spinal cord after nerve injury in the model of peripheral nerve trauma (61). Above all, it is possible that NPY participates in the pathogenesis of osteoporosis. In detail, NPY plays a negative role in the process of osteoporosis.

### Bone Fracture

Bone fracture healing is a multistep and overlapping process involving inflammation, osteogenesis, and angiogenesis (62). Among these processes, the formation of primary bone is a crucial one since it is the key process of fracture healing. Gu et al. focused on patients with traumatic brain injury–fracture-combined injuries and found that the NPY level was increased, accomplished with an increase of bone formation markers, indicating an active role of NPY in fracture healing (46). Sousa et al. generated germline (Y1^−/−^) and osteoblastic-specific Y1R knockout mice to characterize whether Y1R plays a role in fracture healing. The fracture healing process was delayed in the global deletion of Y1R in mice, and this delay is independent from osteoblast-specific Y1R. In Y1R-specific deficient mice, delayed endochondral fracture healing seems to be the result of impaired inflammatory response and cartilage removal since Y1R is widely expressed in neuronal but also in non-neuronal cells, such as immune cells (47). However, Long et al. established an angular fracture rat model and found that regenerating NPY fibers were increased in the early stages and then reduced between 21 and 56 days on the concave side compared with the convex side, suggesting that NPY innervation appears to correlate with the loss of callus thickness in angular fractures (63). Based on the evidence mentioned above, the authors hypothesized that NPY plays an important role in fracture healing, and this role may not be achieved through Y1R. Further study is needed to clarify the underlying mechanism.

### Inflammation

NPY is produced not only by the central and peripheral nervous system but also by immune cells such as macrophages, B cells, neutrophils, and lymphocytes (64). It can cause the activation of immune cell response and induce the release of proinflammatory cytokines including TNF-α or interleukin-6, acting as a potent modulator of the immune responses during inflammation, infection, and autoimmunity (65–67). In animal models of systemic inflammation such as endotoxemia, the expression of NPY in the hypothalamus was slightly increased and positively correlated with the severity of inflammation (68, 69). A cross-sectional design of rheumatoid arthritis (RA) patients found that serum levels of NPY are significantly related to TNF-α levels and disease activity in RA independently of IL-6, TNF-α, or leptin levels (67). In patients with knee osteoarthritis, concentrations of NPY in synovial fluid were gradually upregulated with the severity of pain, suggesting a role for NPY as a putative regulator of joint homeostasis (66). This suggested that NPY plays a crucial role in both systematic and local sites, and often reflected the severity of inflammation.

### Osteoarthritis

As the most common joint disease worldwide, OA is characterized by cartilage degradation, synovial inflammation, subchondral bone remodeling, and osteophyte formation and primarily identified as a non-inflammatory musculoskeletal degeneration (70). Several studies suggest the involvement of NPY in the pathogenesis of OA, and it has already been identified as the major peptide involved both in the generation of pain. NPY concentration in synovial fluid was significantly higher in OA patients compared with controls and positively correlated with pain intensity (66, 71). Kang et al. reported that NPY was overexpressed in human OA cartilage accompanied with increased Y2R expression. Stress stimulus resulted in the sympathetic release of NPY, which in turn promoted the upregulation of NPY and Y2R in articular cartilage and participated in chondrocyte hypertrophy together with cartilage matrix degradation (30). Hernanz et al. demonstrated a significant stimulatory activity of NPY on inflammatory factors such as IL-1β, IL-6, and TNF-α production by whole blood leukocytes from OA patients *in vitro*, which play critical roles in pain in the early stage of OA, indicating a positive effect of NPY in inflammation (72, 73).

### Mood Disorders and Bone Abnormalities

Mood disorders such as chronic stress and depression often have adverse consequences on many organs, including the bone. In view of the negative effects of NPY signaling on bone metabolism mentioned above, NPY activity associated with chronic stress and depression would predict a deleterious influence on bone homeostasis. In multiple sclerosis (MS) patients, autonomic nervous system dysfunction and low BMD are intertwined with some mood disorders such as depression, fatigue, and migraine (74). Higher levels of depression were demonstrated in osteocalcin-deficient mice when compared with wild-type mice, giving evidence to bone signal back to the brain (75). Animal experiments also showed that antidepressants may exhibit clinical efficacy by increasing NPY expression levels (76). However, as a well-described anxiolytic factor, NPY was also reported to exhibit a stress-protective role specifically through Y2 receptors (51). The relationships between NPY and mood disorder and between NPY and bone mass maintenance are intriguing and need further investigations.

## Conclusion

Previous studies have verified that NPY is widely present in the brain and bone tissue and strongly influences bone metabolism through direct and indirect manner. In addition to directly regulating bone formation and resorption, NPY may also participate in bone metabolism by affecting gut microbiota and blood vessel formation. Furthermore, NPY has also been reported to play an intermediary role in autonomic nerve regulation on bone metabolism. As a substance synthesized by multiple places, it will be a challenge to clearly clarify the role of NPY on bone turnover and elucidate the pathophysiology of common bone diseases mentioned above. Also, whether NPY derived from sympathetic nerve endings and osteocytes has different physiological effects remains to be explored. Even though previous studies have shown that NPY participates in bone metabolism, especially in the bone formation process and BMSC fate decision, the effect of NPY on osteoclastogenesis and mood disorder is not fully understood.

In spite of NPY being mostly expressed in the central nervous system, the role of NPY secreted by surrounding tissues, organs, and cell types in bone metabolism and cell signal transduction may be an important future research consideration. Future research on NPY and its receptors will be beneficial for new drug development and identifying new treatments for bone diseases.

## Author Contributions

YZ conceived and designed the manuscript. Q-CC wrote the paper. All authors contributed to the article and approved the submitted version.

## Conflict of Interest

The authors declare that the research was conducted in the absence of any commercial or financial relationships that could be construed as a potential conflict of interest.

## Publisher’s Note

All claims expressed in this article are solely those of the authors and do not necessarily represent those of their affiliated organizations, or those of the publisher, the editors and the reviewers. Any product that may be evaluated in this article, or claim that may be made by its manufacturer, is not guaranteed or endorsed by the publisher.
